# Some aspects of interactivity between endocrine and immune systems required for successful reproduction

**DOI:** 10.1186/s12958-015-0020-5

**Published:** 2015-04-11

**Authors:** Andrea Weghofer, Eric Himaya, Vitaly A Kushnir, David H Barad, Emanuela Lazzaroni-Tealdi, Yao Yu, Yan-Guang Wu, Norbert Gleicher

**Affiliations:** Center for Human Reproduction, New York, NY USA; Department of Obstetrics and Gynecology, Medical University Vienna, Vienna, Austria; Centre Hospitalier de l’Université de Montréal, Montreal, Canada; Foundation for Reproductive Medicine, New York, NY USA

**Keywords:** Immune system activation, Androgens, Testosterone, In vitro fertilization (IVF), Embryo quality, infertility

## Abstract

**Background:**

In successful reproduction, endocrine and immune systems closely interact. We here attempt to further elucidate the relationship between androgen levels, systemic activation of the immune system and reproductive success in infertile women, utilizing 2 distinct infertile patient cohorts.

**Methods:**

In Group 1, we investigated 322 women (ages 38.6 +/− 5.4 years) at initial presentation; in Group 2 125 women undergoing in vitro fertilization (169 IVF cycles, ages 38.9 +/− 5.5 years). In Group 1, we assessed androgens and an immune panel, previously demonstrated to discriminate between activated quiescent immune systems; in Group 2, utilizing the same immune panel, we investigated whether immune system activation relates to embryo quality in IVF cycles.

**Results:**

No individual immune test within the immune panel was associated with androgen levels. The total/free testosterone ratio (TT/FT) was, however, significantly associated with presence of gammopathies (in IgG, IgM, IgA, IgE; P = 0.026). Surprisingly, immune system activation was associated with significantly improved embryo quality (P = 0.008), a finding persistent after adjustment for age and repeat IVF cycles (P = 0.006).

**Conclusions:**

Association of immune system activation with improved embryo quality concurs with previously reported immune activation in association with normal functional ovarian reserve (FOR) and normal androgen levels, while, counter intuitively, hypoandrogenism and low FOR are associated with lack of immune system activation. Mild immune system activation, therefore, likely appears essential for establishment of pregnancy, and may be regulated by androgens.

## Background

Since the implanting embryo is a paternal semi-allograft, successful implantation in the maternal uterus is akin to allogeneic transplantation of a “mini-multiorgan transplant” to the mother. Establishment of pregnancy is, therefore, an immunologically mediated process.

To tolerate the transplant, the mother’s immune system has to “reprogram” itself from rejection to tolerance. How this happens is only incompletely understood [1], but evidence suggests that a normal immune system does this well, while excessively activated immune systems (severely hyper allergenic women or women with inflammation and/or active autoimmunity), do not. Inadequate maternal tolerance then leads to allogeneic maternal immune responses against the implanting fetus and failed implantation and/or pregnancy loss [2].

Since even healthy individuals frequently, demonstrate laboratory evidence suggestive of immune system hyperactivity, normal quiescence versus activation is not well defined. For example, autoimmune abnormalities are frequently found in association with allergies [3] or even in normal patient populations [4,5], yet, even if just sub-clinical, have been associated with reproductive problems, including infertility, miscarriage risk [6,7] and complications of pregnancy, such a premature labor and preeclampsia/ eclampsia [8,9].

We recently noted that women with low functional ovarian reserve (LFOR) at practically all ages are characterized by relative hypoandrogenism [10]. Yet, somewhat counter intuitively, we also discovered that they demonstrated no evidence whatsoever of immune system activation as we initially had hypothesized; yet, young, healthy control patients with normal androgen levels, to some degree did demonstrate evidence of immune system activation [11].

Based on these unexpected findings, we further hypothesized that normal androgen levels, in females approximately equally adrenal and ovarian in origin, depend on mild immune system activation. In absence of activation by an immune system – driven androgen production factor (APF), androgen levels decrease and androgen-dependent early stages of follicle maturation wither [2]. Under this hypothesis, the establishment of pregnancy, thus, would be dependent on mild immune system activation.

While this on first impression may sound like a counter intuitive proposition, various important stages of reproduction, including ovulation and implantation, have already been identified as “inflammatory” processes [12,13].

Here presented study was, therefore, designed to further explore observable effects of immune system activation, and how they may relate to reproductive success.

## Methods

### Institutional review board

Here presented study involved two distinct patient populations, both of which were identified and retrieved from our center’s electronic research data base, where selected medical record data from presenting patients are stored in anonymized fashion for research purposes. Prior to inclusion in this research data bank, patients at our center at initial presentation sign an informed consent, which authorizes the use of their medical record data for research purposes, as long as data remain confidential and the identity of the patient remains protected. Both of these conditions are met when data are extracted from the center’s electronic research database, and the study, therefore, underwent only expedited review and approval from the center’s Institutional Review Board (ER12172012-01, December 18, 2012).

### Patient populations and main outcome measures

This study involved 2 patient populations: Group 1 included 322 consecutive infertile women, who presented to our center for initial diagnostic evaluation at mean age of 38.6 ± 5.4 years. Among those 125 women (Group 2) underwent 169 IVF cycles, with some patients, thus, undergoing more than one IVF cycle. Their mean age was 38.9 ± 5.4 years. Table 1 summarizes patient characteristics for both patient groups, and demonstrates that they in all characteristics were very similar.

**Table 1:** Baseline characteristics of Groups 1 and 2*

	**Group 1**	**Group 2**
	**(n = 322)**	**(n = 125)**
Age (years)	38.6 ± 5.4	38.9 ± 5.4
BMI** (kg/m^2^)	24.0 ± 4.9	24.1 ± 4.6
DHEA (ng/dL)	469.2 ± 289.8	467.9 ± 308.9
DHEAS (μg/dL)	253.5 ± 189.0	371.6 ± 233.4
Total testosterone (ng/dL)	26.0 ± 13.7	33.7 ± 23.5
Free testosterone (pg/mL)	2.0 ± 1.5	2.6 ± 1.8
TSH (μIU/mL)	1.9 ± 1.2	1.9 ± 1.0
Thyroid antibodies	22.0%	16.0%
Anti-adrenal antibodies	0.3%	0%
Anti-ovarian antibodies	9.3%	6.4%
Antinuclear antibodies (ANA)	13.0%	7.2%
Antiphopholipid antibodies (APA)	14.6%	14.4%
Gammopathy	32.0%	36.0%
Immune system activation***	59.0%	59.2%

*Values are presented as means ± standard deviation or as percentages. Patient characteristics did not differ between both groups except for DHEAS (P < 0.001), total testosterone (P < 0.001) and free testosterone (P < 0.01), all three the consequence of DHEA supplementation for women with LFOR between baseline evaluation and IVF cycle start. For further detail, see text.

Percentages among antibodies represent percentages of patients with positive antibody tests.

**BMI, body mass index.

***Defined by presence of at least 1 positive laboratory test.

In order to qualify for Group 1, patients had to have a complete initial data set of laboratory results in the center’s electronic database, which, among immune test, included antinuclear antibody, an antiphospholipid antibody panel, including antibodies to β2-glycoprotein, cardiolipin and phosphatidylserine in IgG, IgM and IgA isotypes, a lupus anticoagulant, an antithyroid antibody panel, including antibodies to thyroid peroxidase, thyroglobulin and thyrotropin receptor, total IgG, IgM, IgA and IgE levels, anti-adrenal antibodies (to 21-hydroxylase) and anti-ovarian antibodies. All of these laboratory tests were obtained via commercially available assays.

Because this panel of tests has in studies been proven effective in discriminating with high sensitivity, though relatively poor specificity, women with immune system activation from those with a quiescent immune system, it is part of our center’s routine initial fertility evaluation at time of presentation. Fully expecting a high degree of false-positivity, any positive finding in the panel is clinically considered to represent immune system activation, and only complete absence of any positive findings is clinically considered a quiescent immune system [11].

In this context the expectation is to achieve high sensitivity but poor specificity of diagnosis. This means that the prevalence of immune system activation will be overestimated; yet, patients considered quiescent will, indeed, with high certainty lack immune system hyperactivity. In this context, statistical associations with immune system activation will, therefore, be clinically diluted by the presence of false positive patients and, therefore, underestimated. So demonstrated statistically significant associations, therefore, have to be considered statistically sound.

Since women with LFOR demonstrate low androgen levels [10], we now also routinely assess androgens in newly presenting patients. Group 1 patients, therefore, also had to have a completed set of androgen values in the center’s research database, including dehydroepiandrosterone (DHEA), DHEA-sulfate (DHEAS), free testosterone (FT) and total testosterone (TT). All of above noted laboratory tests were performed by routine commercial assays, as previously reported, with TT being evaluated with chromatography-tandem mass spectrometry [11].

We then assessed whether in Group 1 patients individual immune parameters or whole immune panels (i.e., thyroid antibodies, antiphospholipid antibodies, total immunoglobulins, etc.) statistically correlated with androgen levels.

As noted above, Group 2 patients, represented as subset 125 infertile women (mean age 38.9 ± 5.4 years), having undergone 169 IVF cycles within 2–4 weeks from last laboratory assessments (Table 1). Group 2 patients were used to assess whether immune parameters statistically correlated with embryo quality in IVF cycles.

Embryo quality was assessed in a total of 777 embryos. Each embryo is individually evaluated under the microscope early in the morning of day-3. Embryo assessments are performed at our center by counting blastomeres, assessing embryo shape, and determining the degree of fragmentation. Embryos were graded based on the following parameters: Excellent or Grade 5, reached 8 cells with <5% fragmentation; Good or Grade 4, reached 6–8 cells with ≤5% fragmentation; Average or Grade 3, 6–8 cells with 5-20% fragmentation; Poor or Grade 2, reached 4–8 cells with 20-40% fragmentation; Very poor or Grade 1, 4–8 cells, with >40% fragmentation.

### Statistical analyses

To investigate the effects androgen levels on immune system activation, generalized linear mixed-effects models (GLME) were used. GLME were also used to study the effects of immune system activation on embryo quality. The models were then controlled for age and repeated cycles in Group 2. Analyses were conducted using SAS 9.2. P-values <0.05 were considered statistically significant. All original data are available to investigators from CHR’s electronic data depository by contacting David H. Barad, MD, MS at dbarad@thechr.com.

## Results

### Group-1 results

Patient characteristics for this group of infertility patients are summarized in Table 1. As the table demonstrates, their mean age was 38.6 ± 5.4 years. Their body mass index (BMI) was 24.0 ± 4.9 kg/m^2^.

It is also important to note that except for DHEAS and testosterone levels, none of the other patient characteristics differed between Groups 1 and 2. DHEA and testosterone levels increased in Group 2 patients because our center routinely supplements women with LFOR prior to IVF cycle start (see below).

Looking at the patients’ individual levels of DHEA, DHEAS, TT, FT and TT/FT-ratios, none was individually associated with laboratory evidence of thyroid autoimmunity, presence of elevated antiphospholipid antibodies, antinuclear antibodies, anti-adrenal autoimmunity or anti-ovarian antibodies. TT/FT ratios were, however, statistically associated with gammopathies, either abnormally high or low total immunoglobulin levels in all isotypes (P = 0.026; Table 2).

**Table 2:** **Statistically significant associations**

**Associations**	**Significance**
TT/FT with presence of gammopathies (IgG, IgM, IgA, IgE)	P = 0.026
Immune system activation* with embryo quality	P = 0.008
After adjustments for age and repeat cycles	P = 0.006

TT/FT, total/free testosterone ratio.

*As defined in Table 1.

### Group-2 results

Even more interesting, however, were observations in Group 2, where the mean age was almost identical to Group-1 patients (38.9 ± 5.4 years, Table 1). Out of 777 embryos, 353 (45.4%) were of good, 337 (43.4%) were of fair and 87 (11.2%) were of poor quality. Defining immune system activation as presence of one or more immune abnormalities, evidence for female immune system activation was associated with significantly better embryo quality than absence of such evidence (P = 0.008). This association, indeed, persisted even after adjustments for female age and repeat IVF cycles (P = 0.006), strongly suggesting that mild immune system activation offers benefits in regard to embryo quality. The observed improvement in embryo quality was primarily the consequence of higher embryo grades (3.7 ± 0.05 vs. 3.4 ± 0.07; P = 0.002).

## Discussion

Here presented data on random female infertility patients confirm the previously reported association between androgen levels and immune system activation [11], this time, however, even more specifically demonstrating that abnormalities in total immunoglobulin levels are statistically associated with TT/FT ratios.

This is a potentially interesting new observation since TT/FT ratios are to a large degree dependent on sex hormone binding globulin (SHBG), as defined by the Free Androgen $\mathrm{Index}\ \left(\mathrm{F}\mathrm{A}\mathrm{I} = 100 \times \frac{\mathrm{TT}}{\mathrm{SHBG}}\right)$ [14]. While we, unfortunately, in this study did not have data on SHBG, these observations potentially link immunoglobulin concentrations via androgen levels to SHBG levels, as androgens are known to decrease SHBG [15,16], thus resulting in more of bioactive FT becoming available.

The association of TT/FT ratios and immunoglobulin abnormalities supports the previously reported association between mild immune system activation [11], normal ovarian function and normal androgen levels; while women with abnormally low ovarian function not only lack evidence of immune system activation but also demonstrate comparatively low androgen levels [10].

We, therefore, hypothesized about existence of a potential “immunologic” androgen production factor (APF), responsible for maintaining appropriate androgen levels for normal female fertility, which may be potentially increased in association with polycystic ovarian syndrome (PCOS) and decreased in women with LFOR ([11] and reviewed in [2]). Here presented data offer further support for such a hypothetical APF.

A possible association between immune system activation and embryo quality has never before ben reported. This observations in Group 2-women is, therefore, likely even more significant. It is not only statistically very robust but even held up after adjustment for age and number of earlier IVF cycles, suggesting that it is independent of female age and, at least to a degree, independent of severity of infertility.

Combined, these observations, therefore, suggest that a mild form of immune system activation may represent a highly important theme throughout all stages of successful reproduction, starting with egg/embryo quality, as here reported for the first time.

This concept is not necessarily new: For example, Jabbour et al. pointed out the importance of inflammatory pathways throughout reproductive physiology, from ovulation, to menstruation, implantation and onset of labor, all now increasingly perceived as inflammatory processes [17]. Here presented study suggests that creation of good embryo quality now also may have to be added as a process dependent on inflammatory pathways.

All of these observations also explain the close relationship and interplay between endocrine and immune systems, recently increasingly addressed in the literature [2,9].

How an activated immune system could beneficially affect egg and embryo quality remains to be established. Androgens, however, allow for a hypothesis: Recently reported animal models [18,19] and human clinical data ([20] reviewed in [21]), suggest that androgens are essential for early stages of follicle maturation. Improving egg/embryo quality via androgen supplementation in women with LFOR and with low androgen levels, therefore, appears to increase pregnancy chances [20], to reduce aneuploidy in embryos [22] and, therefore, miscarriage rates [23].

Androgens are, however, also strongly anti-inflammatory, and are, therefore, for that purpose clinically, for example, utilized in autoimmune disease [24]. The previously noted hypothetic immune system-derived APF with the ability to increases androgen levels, thus, potentially can create a feed-back loop by inducing anti-inflammatory androgens which, in turn, keep a low-level of inflammatory immune system activation in check.

In conclusion, here presented results add to an increasing volume of data pointing toward significant interaction between endocrine and immune systems in establishing reproductive success. Albertini recently described the ovaries as immunological “hot-spots” [25] based on the recognition that increasing numbers of fertility-associated genes in the ovary also have immunological functions. It increasingly appears that other endocrine organs may deserve a similar designation. Indeed, the brain and other endocrine organs like the adrenals, increasingly appear to represent locations where endocrine and immune systems almost merge into one immune/endocrine system (2). For successful reproduction the flawless function of this united system appears increasingly essential.
